# Objective assessment, repeatability, and agreement of shoulder ROM with a 3D gyroscope

**DOI:** 10.1186/1471-2474-14-72

**Published:** 2013-02-26

**Authors:** Bilal Farouk El-Zayat, Turgay Efe, Annett Heidrich, Robert Anetsmann, Nina Timmesfeld, Susanne Fuchs-Winkelmann, Markus Dietmar Schofer

**Affiliations:** 1 Department of Orthopaedics and Rheumatology, University Hospital Marburg, Baldingerstrasse, Marburg, 35033, Germany; 2 Institute for Medical Biometry and Epidemiology, Philipps University Marburg, Bunsenstraße 3, Marburg, 35037, Germany

**Keywords:** Repeatability, Precision, Shoulder motion, Objective assessment, Dynaport, Gyroscope

## Abstract

**Background:**

Assessment of shoulder mobility is essential for diagnosis and clinical follow-up of shoulder diseases. Only a few highly sophisticated instruments for objective measurements of shoulder mobility are available. The recently introduced DynaPort MiniMod TriGyro ShoulderTest-System (DP) was validated earlier in laboratory trials. We aimed to assess the precision (repeatability) and agreement of this instrument in human subjects, as compared to the conventional goniometer.

**Methods:**

The DP is a small, light-weight, three-dimensional gyroscope that can be fixed on the distal upper arm, recording shoulder abduction, flexion, and rotation. Twenty-one subjects (42 shoulders) were included for analysis. Two subsequent assessments of the same subject with a 30-minute delay in testing of each shoulder were performed with the DP in two directions (flexion and abduction), and simultaneously correlated with the measurements of a conventional goniometer. All assessments were performed by one observer. Repeatability for each method was determined and compared as the statistical variance between two repeated measurements. Agreement was illustrated by Bland-Altman-Plots with 95% limits of agreement. Statistical analysis was performed with a linear mixed regression model. Variance for repeated measurements by the same method was also estimated and compared with the likelihood-ratio test.

**Results:**

Evaluation of abduction showed significantly better repeatability for the DP compared to the conventional goniometer (error variance: DP = 0.89, goniometer = 8.58, p = 0.025). No significant differences were found for flexion (DP = 1.52, goniometer = 5.94, p = 0.09). Agreement assessment was performed for flexion for mean differences of 0.27° with 95% limit of agreement ranging from −7.97° to 8.51°. For abduction, the mean differences were 1.19° with a 95% limit of agreement ranging from −9.07° to 11.46°.

**Conclusion:**

In summary, DP demonstrated a high precision even higher than the conventional goniometer. Agreement between both methods is acceptable, with possible deviations of up to greater than 10°. Therefore, static measurements with DP are more precise than conventional goniometer measurements. These results are promising for routine clinical use of the DP.

## Background

Of all the joints in the human body, the shoulder joint has the highest range of motion (ROM). Hence, objective assessment of shoulder joint mobility, especially after conservative or operative therapy, is critical. Moreover, correct assessment of shoulder mobility is crucial for grading of several clinical shoulder scores (Constant-Score, Rowe-Score, Simple Shoulder test).

Only a few highly sophisticated instruments are available for objective measurement of shoulder mobility. In daily practice, they are time consuming, complicated, expensive, or not applicable [1,2]. Human motion analysis systems require cable wires, synchronization, external references, mounting sensors to the subject, among others. On the other hand, conventional goniometers as standard measurement tools can only measure joint angles statically, and have low reliability and precision between individual instruments [3]. This error is compounded in shoulder patients with decreased mobility [4].

A new small and handy three-dimensional (3D) gyroscope called DynaPort MiniMod TriGyro ShoulderTest (DP) (McRoberts Inc., The Hague, Netherlands) [5] was designed to assess upper extremity function. The DP has more applications than a conventional goniometer: it can measure complex shoulder movements with rotation, and 3D velocity. Moreover, measurements can be performed continuously for up to 72 hours (e.g., for monitoring shoulder motion during postoperative home training or at work). This device has been validated in a previous laboratory study, showing good reproducibility of measurements [6]. We aimed to assess the precision (repeatability) and agreement of the DP [7] in human subjects for static motions, compared to the conventional goniometer.

The study design was approved by the Ethical Committee of Philipps-University Marburg, reference no. 154/08.

## Methods

### Device

#### DynaPort MiniMod TriGyro ShoulderTest

**Figure 1:**
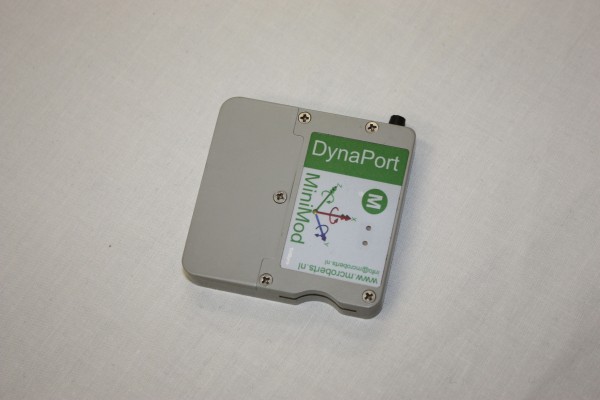
Three-dimensional gyroscope DynaPort MiniMod TriGyro ShoulderTest.

The DP is a small box (62 × 41 × 18 mm, weighing 53 g) containing three gyroscopic sensors (Figure 1). The three DP gyroscopes measure rotation and angular velocity, which can then be converted to angle by a specific mathematical algorithm. The only preparation needed to calculate angles is to teach the device the axis of the shoulder joint by performing a calibration procedure in two directions (e.g. flexion and abduction). The DP is fixed to the distal upper arm with a flexible belt. Subsequently, calibration is executed by consecutive movement of the arm in one plane up to an angle of at least 40° (abduction and flexion). The proper assessment is then performed with five repetitions in each direction. After using matrix algebra and goniometric operations, movement is expressed in elevation and simultaneous internal- and external rotation of the upper arm as a mean value of these five repetitions. Because of its small size and battery operation, assessment is possible anywhere for up to 72 continuous hours. The raw data is stored on a commercially available secure digital (SD) card. Using special software (MiRA®, McRoberts Inc., The Hague, Netherlands), measurement calibration is checked and can be adapted. In a second step, all results can be displayed and evaluated. Digital encryption of the data can also be performed, and uploaded to the company’s website for analysis. A subsequent PDF file with relevant processed data is sent back within a few minutes via an automated e-mail.

**Figure 2:**
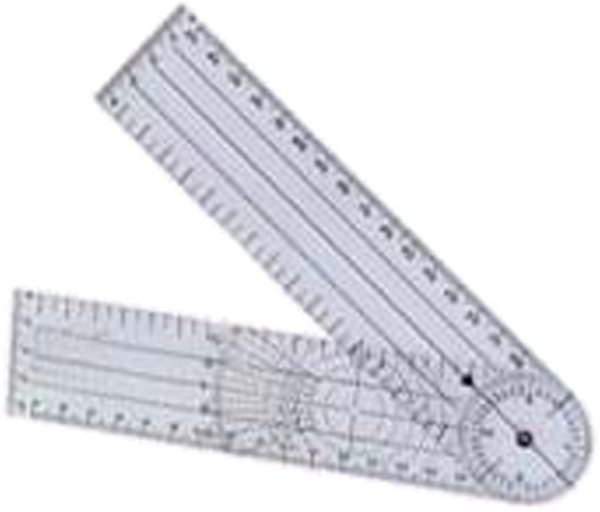
Conventional goniometer.

For direct comparison of measurements, a conventional goniometer was used (Figure 2).

### Subjects and data acquisition

Adult subjects, students and physiotherapists 18 years and older were recruited on a voluntary basis. Twenty-one healthy humans (aged 20–59 years, 12 female and 9 male), agreed with written informed consent to take part in this study. Patients with previous surgery or pain in the shoulders and spine were excluded. Subjects from different sexes and ages were selected, to make validity meaningful. All subjects were evaluated in the same institution, with the same instruments, and by the same observer.

Different studies have shown that the cervical and thoracic spine has an influence on shoulder movement [8]. This error was minimized by fixing the trunk and spine by placing them against a wall during the examination. The primary shoulder for examination purposes was defined by coin toss. For direct comparison, we fixed the DP at the lower portion of the upper arm. Then, the SD memory card was activated. Subsequently, the DP was calibrated with a standard single flexion and abduction movement in order to adjust the gyroscopes in the three dimensions. The start position is considered the neutral position, at 90° elbow flexion. As the calibration procedure is highly sensitive to disturbances and noise in the signal, it is very important to perform movements only in one axis. The manufacturer recommends performing all calibration movements higher than 40° for better precision.

The relevant testing began with five repetitions of abduction with simultaneous measurement by the DP and conventional goniometer. The DP system generates a mean value of the five repetitions. Flexion movements were examined in the same manner. Subsequently, the DP system was demounted and remounted on the contralateral arm, and this procedure was repeated after a 30 minute break.

This study was approved by our institutional Ethics Committee, according to the Declaration of Helsinki.

### Statistical analysis

#### Precision

Precision is defined by a measurement’s repeatability, as the variation between repeated measurements, when all conditions are kept constant (i.e., by using the same instrument and operator, and repeating measurements over a short time period). In the present study, the repeatability of both assessment methods was defined as the variance between two repeated measurements of flexion and abduction. Linear-mixed models were used according to Carstensen [9]. From these models, precision was estimated by error variance for both methods. Subsequently, variance was compared within these models by likelihood-ratio tests.

#### Agreement

It is unlikely that measurements obtained by different methods will agree exactly. Agreement describes the degree to which measurements between the conventional goniometer and the DP are consistent. The mean differences (95% limits of agreement) in measurements performed by the conventional goniometer and DP were determined and illustrated by Bland-Altman plots. Differences between methods were graphically demonstrated.

A p-value of less than 0.05 was considered statistically significant. All statistical analysis was performed using the R program (http://www.r-project.org; version 2.12.1; MethComp).

To determine the number of shoulders required, a power analysis was performed. For simplification, independency between variance estimations was assumed. For 20 subjects, a total sample size of 40 shoulders, a variance ratio of 3.7 could be detected with a power of 80% at a double-sided significance level of 5%.

## Results

### Calibration

**Figure 3:**
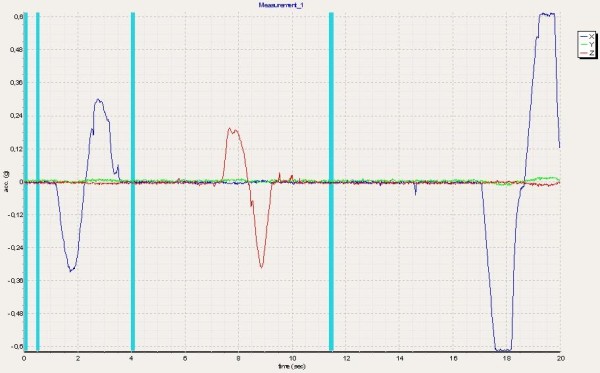
Calibration-check by MiRA®-software: left for flexion and right for abduction.

The quality of calibration was evaluated by special software (MiRA®, Figure 3). The registered course of movement was visualized by two-dimensional (2D) graphs. Another option to evaluate the quality of calibration was based on the reports sent back by the company’s data sheet. The quality of calibration based on the amount of rotational error was presented on a numeric scale. In an optimal case, the calibration movement would be performed without internal or external rotation.

The results of the calibration procedure in the presented assessments were satisfactory with a mean of 9.8 (range: 8.0 – 10.0). If the calibration of a measurement was below 8, the assessment was repeated.

### Measurements on subjects

The overall results of the comparative measurements in the 21 subjects are shown in Table 1. The abduction ROM was similar for both methods and ranged from approximately 115–200°. Comparing different assessment methods, DP produces higher maximum values, whereas mean values appear to be equal. The evaluation of flexion showed similar results for both the DP and conventional goniometer. Subjects showed a mean higher ROM in the 2^nd^ measurement for both directions. Mean standard deviations for flexion movements were obviously lower than for abduction movements, and were higher in the repetition series as well. Comparing different sides, lower standard deviations were found for left-sided movements, for both abduction and flexion, as well as in the first and second series. If the ROM on the examined side was low, it was same on the contralateral side, and vice versa.

**Table 1:** Descriptive results separated for abduction and flexion for each method with mean values for all subjects as well as standard deviations

			**Abduction**		**Flexion**	
**Device**	**Measurement**	**Shoulder**	**mean (sd)**	**Range**	**mean (sd)**	**Range**
**conventional Goniometer**	**1**^**st**^	left	163 (17.4)	122 -- 184	171 (9.88)	152 -- 186
		right	164 (17.1)	119 -- 186	166 (15.6)	129 -- 185
	**2**^**nd**^	left	165 (16.5)	128 -- 182	172 (8.89)	156 -- 186
		right	165 (17.8)	116 -- 184	171 (15.5)	130 -- 184
**Dynaport**	**1**^**st**^	left	162 (19.6)	119 -- 194	171 (11.5)	151 -- 190
		right	163 (19.1)	118 -- 197	166 (16.1)	127 -- 188
	**2**^**nd**^	left	164 (18.3)	121 -- 194	171 (11.7)	150 -- 190
		right	163 (20.7)	114 -- 201	170 (15.0)	133 -- 188

Comparing DP and conventional goniometer in both motion directions (flexion and abduction) showed an average difference of less than 2°.

### Precision and agreement

#### Flexion movement

**Figure 4:**
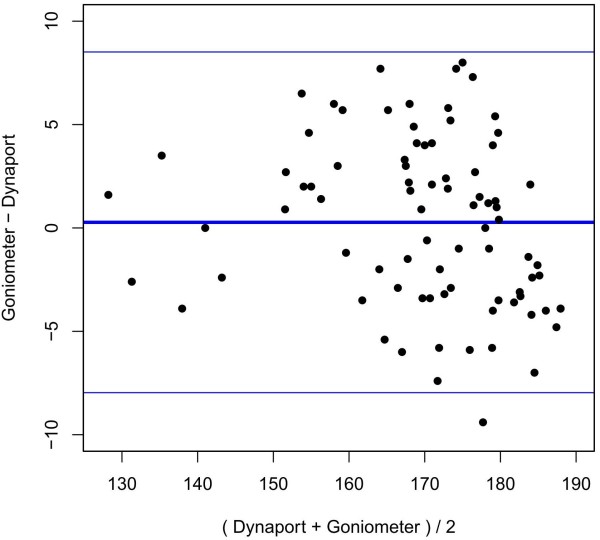
**Bland-Altman-Plot for flexion.** x-axis: mean value of ROM, y-axis: difference between conventional goniometer and DP in °. Thick blue line: mean difference between conventional goniometer and DP. Thin blue lines: lower and upper 95% limits of agreement.

For measurements of the same maximal flexion at the same time, a mean difference of 0.27° was present between the conventional goniometer and the DP. The 95% limit of agreement for two subsequent measurements with a different instrument (either conventional goniometer or DP) ranges from −7.97° to 8.51° (Figure 4). While the estimated precision of the DP (measurement error variance of 1.52) was greater than that of the conventional goniometer (measurement error variance, 5.94), the difference was not statistically significant (p = 0.09).

#### Abduction movement

**Figure 5:**
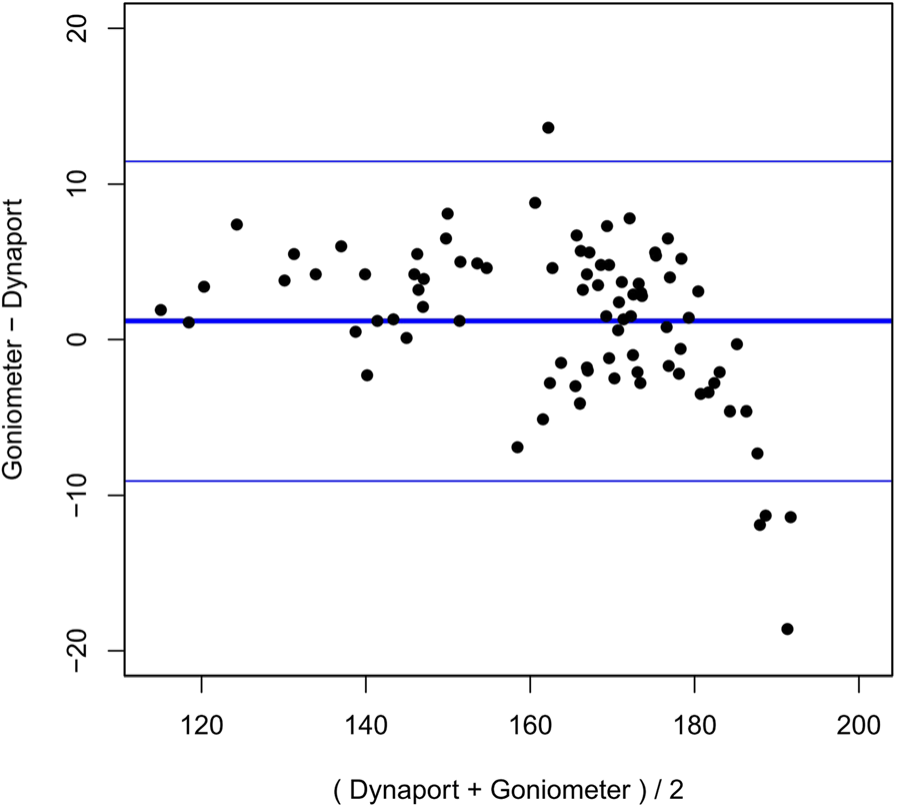
**Bland-Altman-Plot for abduction.** x-axis: mean value of ROM, y-axis: difference between conventional goniometer and DP in °. Thick blue line: mean difference between conventional goniometer and DP. Thin blue lines: lower and upper 95% limits of agreement.

For measurements of the same maximal abduction at the same time, a mean difference of 1.19° was found between the conventional goniometer and the DP. The 95% limit of agreement for two subsequent measurements with each instrument ranged from −9.07° to 11.46° (Figure 5). The precision of the DP was estimated by the error variance (0.89) and was significantly (p-value: 0.025) more precise than the conventional goniometer (error variance, 8.58). No systematic difference could be observed.

## Discussion

Objective assessment of shoulder function is essential in treatment and evaluation of different conditions. Most existing instruments for objective evaluation of shoulder function are not applicable in clinical practice [1,2] and only a few authors compared these instruments for shoulder ROM [10-12]. In this study, we evaluated the precision of a new multifunctional tool, which we have presented recently [6] for two movement directions compared to a conventional goniometer. Results showed significantly improved precision with the DP (in abduction) compared to the conventional goniometer. For flexion, no difference in precision was observed.

Isolated joint movements are difficult to perform. Shoulder joint abduction greater than 90° typically shows movement out of the frontal plane with a tilt of the thorax. Clinical perception therefore cannot be fully captured with a 2D conventional goniometer assessment. Instruments that can recognize the 3D joint position by calculating a projection angle could be a good solution.

Greenfield et al. demonstrated that internal and external rotation of the shoulder shows greater repeatability than flexion and extension [13]. The repeatability of shoulder extension and flexion testing is best when performed in a neutral position, as repeatability decreases with shoulder abduction [14]. This seems to be due to the higher portion of 3D movements, especially in abduction, due to the anteverted position of the glenoid and the shoulder plane. Furthermore, trunk stabilization is important for repeatable measurements [15]. We were able to minimize error during these movements by correct and standardized positioning of the subjects against a wall. Moreover, we know that assessments in obese patients have lower accuracy because the neutral position cannot easily be reached, and movements start in abduction position [15]. This point was not relevant in our study, as the maximal range of motion was the focus, not the starting position.

### Calibration

Calibration movements (flexion and abduction) should be performed in a rectangle in order to obtain the highest precision. The manufacturer’s recommendation for calibration suggests that the velocity of movements should not be slow, without defining it. In the present study, no relevant differences of precision of calibration were found in subjects of different ages.

It should be mentioned that in regular settings, calibration of the DP cannot be checked by the observer. It is intended by the manufacturer that all measured data are uploaded to a central internet server and returned via e-mail after analysis. Insufficient calibration is first reported, when assessments and data uploads are completed. In that case, complete assessments must be repeated. In this study setting, the analysing software was provided for direct analysis in the laboratory, but was viewable after the end of the examination. An optical feedback control on the device that would immediately show quality of calibration (good, modest, poor) might be a practical solution.

### Precision and agreement

There are several potential sources of error that can threaten the credibility of assessments: machine linked inconsistencies, such as differences between tests on the same machine during operation or calibration. These were excluded as in previous studies, by always using the same device so that the validity of the DP system could be proven [6]. If subsequent assessments were performed with the same device, only a difference of up to 2.2° was recognized. Alternatively, subject variations (motivation, pain, fatigue, etc.) when performing repeated or multiple measurements, as well as testing procedure errors (poor/inconsistent stabilization) can occur. These factors are extraordinarily difficult to assess quantitatively and are hard to interpret. Further sources of error could be protocol variations (e.g., different rest periods), intra- and [16] inter-[17] tester reliability (difference between examiners) or data processing factors (e.g. software). All avoidable factors were excluded in the study design. Inter-tester bias [17] was excluded, as the same observer performed all measurements. The test and retest protocol was the same, with one observer and same health status of the subjects, as trials were repeated on the same day. Fatigue was excluded, as a break between both assessments was included to rest the muscles. Moreover the DP is a light instrument, so that muscle power deficit is not relevant. Furthermore, healthy subjects without shoulder (or spine) complaints were selected, to avoid fatigue or pain-related bias. Subject motivation could be seen by the higher maximum values for ROM in the retest series.

Testing procedure errors, such as poor or inconsistent stabilization of the DP on the subject’s arm, or different elbow positions, could explain the variances in part. Further sources of error could be related to data processing (software error) as well as different distances from the centre of rotation in different lengths of arms. The analysing software had not changed over the period of the study. A possible software error would result in a constant bias, which was unlikely considering the fact that the measurements agreed quite well with the measurements performed with the manual goniometer. Other influences on measurement were not observed, as different distances from the centre of rotation did not show a negative influence in earlier trials [6]. This means that, due to gyroscope rotation in all three planes, the positioning of the DP and its orientation in space appears to always be correct if calibrated accurately.

This instrument allows joint angle measurements with at least the same precision as the standard conventional goniometer method, while capturing complex and dynamic movements at any time.

## Conclusion

The benefit of the DP is its easy and inexpensive application, which makes it affordable for physiotherapists and physician offices for objective evaluation of shoulder mobility during therapy.

Future studies must incorporate patients with diseased shoulders to determine the reliability and validity of this instrument in this cohort. Further scientific interest could include optic and video monitoring systems [18] from kinematic studies in comparison to the DP. Moreover, its practicability in day-to-day use must be clarified.

Future implementations of the DP could include real-time applications or biofeedback (balance control, limitation of ROM in shoulder rehabilitation, etc.).

In summary, the DP is easy to apply and highly user friendly. The DP has good repeatability in measurements of shoulder ROM, with better results than conventional goniometers. Especially due to its simple handling and short duration of tests, this method is applicable in clinical practice and can objectively measure the functional disability of the shoulder joint and the results of interventions.

## Competing interests

The authors declare that they have no competing interests.

However, McRoberts BV Inc. provided the DynaPortTriGyro Shoulder Test System and software for the study for free. McRoberts BV Inc. provided no funding and had no input into the design of the study.

## Authors’ contributions

BFE participated in the study design, carried out the study, interpreted the results, and drafted the manuscript. AH and RA carried out the study and participated in interpretation of the results as well as in the draft of the manuscript. NT set up the protocol and performed statistical data analysis, and participated in interpretation of the results as well as in the draft of the manuscript. SFW participated in the study design and interpretation of the results. TE and MDS participated in the study design, interpretation of the results and draft of the manuscript. All authors read and approved the final manuscript.

## Pre-publication history

The pre-publication history for this paper can be accessed here:

http://www.biomedcentral.com/1471-2474/14/72/prepub

## References

[B1] Bernmark E Wiktorin C A triaxial accelerometer for measuring arm movements Appl Ergon 2002 33 6 541 547 10.1016/S0003-6870(02)00072-8 12507338

[B2] Uswatte G Miltner WH Foo B Varma M Moran S Taub E Objective measurement of functional upper-extremity movement using accelerometer recordings transformed with a threshold filter Stroke 2000 31 3 662 667 10.1161/01.STR.31.3.662 10700501

[B3] Terwee CB de Winter AF Scholten RJ Jans MP Deville W van Schaardenburg D Bouter LM Interobserver reproducibility of the visual estimation of range of motion of the shoulder Arch Phys Med Rehabil 2005 86 7 1356 1361 10.1016/j.apmr.2004.12.031 16003664

[B4] de Winter AF Heemskerk MA Terwee CB Jans MP Deville W van Schaardenburg DJ Scholten RJ Bouter LM Inter-observer reproducibility of measurements of range of motion in patients with shoulder pain using a digital inclinometer BMC Musculoskelet Disord 2004 5 18 10.1186/1471-2474-5-18 15196309 PMC434511

[B5] Van Hees VT Slootmaker SM De Groot G Van Mechelen W Van Lummel RC Reproducibility of a triaxial seismic accelerometer (DynaPort) Med Sci Sports Exerc 2009 41 4 810 817 10.1249/MSS.0b013e31818ff636 19276852

[B6] El-Zayat BF Efe T Heidrich A Wolf U Timmesfeld N Heyse TJ Lakemeier S Fuchs-Winkelmann S Schofer MD Objective assessment of shoulder mobility with a new 3D gyroscope–a validation study BMC Musculoskelet Disord 2011 12 168 10.1186/1471-2474-12-168 21777447 PMC3151225

[B7] Faber HvH H van Ipenburg S van Lummel RC Measurement of the Elevation and Rotation of the Humerus using a 3D Accelerometer Congress of the Dutch Society of Arthroscopy 2006 Ermelo, Denmark: Oral presentation during the Congress of the Dutch Society of Arthroscopy

[B8] Theisen C van Wagensveld A Timmesfeld N Efe T Heyse TJ Fuchs-Winkelmann S Schofer MD Co-occurrence of outlet impingement syndrome of the shoulder and restricted range of motion in the thoracic spine–a prospective study with ultrasound-based motion analysis BMC Musculoskelet Disord 2010 11 135 10.1186/1471-2474-11-135 20587014 PMC2903509

[B9] Carstensen B Simpson J Gurrin LC Statistical models for assessing agreement in method comparison studies with replicate measurements Int J Biostat 2008 4 1 Article 16 22462118 10.2202/1557-4679.1107

[B10] Barnett ND Duncan RD Johnson GR The measurement of three dimensional scapulohumeral kinematics–a study of reliability Clin Biomech (Bristol, Avon) 1999 14 4 287 290 10.1016/S0268-0033(98)00106-5 10619117

[B11] Hayes K Walton JR Szomor ZR Murrell GA Reliability of five methods for assessing shoulder range of motion Aust J Physiother 2001 47 4 289 294 11722295 10.1016/s0004-9514(14)60274-9

[B12] Mullaney MJ McHugh MP Johnson CP Tyler TF Reliability of shoulder range of motion comparing a goniometer to a digital level Physiother Theory Pract 2010 26 5 327 333 10.3109/09593980903094230 20557263

[B13] Greenfield BH Donatelli R Wooden MJ Wilkes J Isokinetic evaluation of shoulder rotational strength between the plane of scapula and the frontal plane Am J Sports Med 1990 18 2 124 128 10.1177/036354659001800202 2343977

[B14] Chan KM Maffulli N Nobuhara M Wu JJ Shoulder instability in athletes. The Asian perspective Clin Orthop Relat Res 1996 323 106 112 8625565 10.1097/00003086-199602000-00014

[B15] Frisiello S Gazaille A O’Halloran J Palmer ML Waugh D Test-retest reliability of eccentric peak torque values for shoulder medial and lateral rotation using the Biodex isokinetic dynamometer J Orthop Sports Phys Ther 1994 19 6 341 344 8025574 10.2519/jospt.1994.19.6.341

[B16] Molczyk L Thigpen LK Eickhoff J Goldgar D Gallagher JC Reliability of testing the knee extensors and flexors in healthy adult women using a Cybex II isokinetic dynamometer J Orthop Sports Phys Ther 1991 14 1 37 41 18796831 10.2519/jospt.1991.14.1.37

[B17] Michael T Gross GMH Phillips CN Ann Wray J Intramachine and intermachine reliability of the Biodex and Cybex® II for knee flexion and extension peak torque and angular work J Orthop Sports Phys Ther 1991 13 6 329 335

[B18] Raiss P Rettig O Wolf S Loew M Kasten P Range of motion of shoulder and elbow in activities of daily life in 3D motion analysis Z Orthop Unfall 2007 145 4 493 498 10.1055/s-2007-965468 17912671

